# Competing health policies: insurance against universal public systems

**DOI:** 10.1590/1518-8345.1074.2668

**Published:** 2016-03-04

**Authors:** Asa Ebba Cristina Laurell

**Affiliations:** 1 PhD, Researcher

**Keywords:** Health Policy, Insurance, Health, Unified Health System

## Abstract

Objectives: This article analyzes the content and outcome of ongoing health reforms in Latin America: Universal Health Coverage with Health Insurance, and the Universal and Public Health Systems. It aims to compare and contrast the conceptual framework and practice of each and verify their concrete results regarding the guarantee of the right to health and access to required services. It identifies a direct relationship between the development model and the type of reform. The neoclassical-neoliberal model has succeeded in converting health into a field of privatized profits, but has failed to guarantee the right to health and access to services, which has discredited the governments. The reform of the progressive governments has succeeded in expanding access to services and ensuring the right to health, but faces difficulties and tensions related to the permanence of a powerful, private, industrial-insurance medical complex and persistence of the ideologies about medicalized 'good medicine'. Based on these findings, some strategies to strengthen unique and supportive public health systems are proposed.

## Introduction

Health as a right has become a topic of debate and political-ideological struggle. This happens because there are currently two different ways of understanding what is meant by a universal right to health. On the one hand, the concept of Universal Health Coverage (UHC) is based on the assurance that it covers a limited package of services. On the other hand, there is the concept of attaining this through a free, single, public health system and ensuring equal access for all, in light of the same need, known as the Unified Health System (Sistema Único de Salud - UHS). 

The UHS has worldwide hegemonic claims and is supported by supranational institutions such as the World Bank and even the World Health Organization (WHO), philanthropic organizations such as the Rockefeller Foundation, and the new spaces of the transnational oligarchy, such as the World Economic Forum. This commodifying and focused conception is influenced by neoliberal discourse and represents a kind of "unique thinking" in the field of health. 

The other concept is promoted by the progressive governments as well as left-wing forces and parties. In fact, this approach is the only one that meets the definition of the universal right to health, which means recognizing the intrinsic and equal worth of every human being and ensuring access to needed health services without discrimination. In practical terms, it is guided by health needs and is part of the construction of social citizenship. It expresses a worldview in which the collective well-being, dignity and human life are basic values.

## The complexity of the health field

To understand the tensions in health policies, it is necessary to recognize the complexity of this area. It is important to examine its various dimensions to clarify how tensions and contradictions arise, in order to create ways to address them. 

First, we must assume that health is a scientific-technical object with specialized and complex content in which the professional medical-scientific view dominates the comprehensive social and sympathetic view. Medicine presents itself, in this sense, as a topic for experts. There is also a widespread ideology in society about what 'good medicine' is. This is understood as that which has high technological density, uses next-generation drugs and provides good hospitality. Not only is this the view of physicians and other health professionals, but it tends to be shared by the public and politicians. 

Consequently, they both expect tangible and relatively quick results using skilled and technically competent care for patients under this model; this is a model which also leads to health being conceived of as a consumable good rather than an area of rights and citizenship^(^ 1 ^)^. 

In any country, the health sector is among the most significant business activities, accounting for between five and 18% of the gross domesting product (GDP). Although a considerable part of the costs is in human resources, public health institutions additionally require large budgets to purchase pharmaceuticals, other supplies, equipment, and for maintenance. This exposes them to corrupt practices, influence peddling and diversion of resources. 

This economic weight turns medical activities into an important area for income generation and capital accumulation. In the last decade, health insurance plans have increased their importance within the medical-industrial complex, consolidating the financial capital in this sector and the rest of the economy. Thus, important economic interests are present with political power and lobbying capacity in the industry. 

Finally, unlike education that has a permanent presence in the life of population, health tends to be a temporary concern related particularly to the onset of a disease or life threatening condition. 

This complexity creates contradictions, tensions and temptations in health policies with opposition to the progressive, legal policies of social democratic and neoliberal states.

## Neoliberal health policy and health insurance

Latin America stands out as a testing ground for neoliberal health policy in two stages: the commodification-subsidiarity, and the UHC. In 1993, the plan for neoliberal health action, "Investing in Health was launched on an international basis^(^ 2 ^)^", but Chile had already applied its reform in 1981/1982; in Colombia the Law 100 was also approved with this orientation, in 1993. However, almost no Latin American country has been spared from this type of reform over the past two decades, with the invariable weakness of their public health systems. 

The neoliberal reform basically challenges the idea of heath as a human and social right, and moves toward its commercialization. This policy is based on neoclassical economics with its premise that the market is the best distributor of resources, and that competition improves quality and abates costs^(^ 3 ^)^; a premise that has never been proven in health. It redefines, on the one hand, the responsibilities of the State, market and family/individual with regard to health and, on the other hand, it redefines the words 'private property' and 'public goods'^(^ 4 ^)^, which have created serious epistemological confusion. 

The new distribution of responsibilities places the private market in the center, whether these are for-profit companies or families/individuals, while the state's role is subsidiary and only serves those proven to be poor, in targeted health program packages which are cost-effective and restricted, and produce 'public goods'^(^ 5 ^)^ . 

In its new definition, 'private property' is that which is consumed by individuals; a category that includes individual health care. In this situation, the UHC model focuses on individuals. This means that the actions of public health or those aimed at the community belong to another category. 

The 'public goods', which must be borne by the government, are defined as those which are characterized by 'non-inclusiveness' (cannot exclude anyone from consumption) and 'non-rivalry' (consumption by someone prevents consumption from another). Although they do not strictly conform to the above criteria, goods with 'large externalities' are located in this group. These are directed to the individual but also protect the community, namely, essentially public health actions such as epidemiological surveillance or vaccines. Another staple element in the neoliberal reform is the decentralization of services which, in practice, is equivalent to the decentralization of the political responsibility of the central state, at the request of lower-level political and administrative authorities^(^ 6 ^)^. 

The initial neoliberal health policy faced ideological complications, political protests and economical exclusions, such as the rest of the social and economic policies. This forces neoliberal governments to push for a second reform or modernization of the state^(^ 7 ^)^ in which the proposal from the UHC^(^ 8 ^)^ is located. It differs from the first health reform that emphasized the strict separation of functions between: regulation by the State; public or private fund/purchasing services management; private or public provision of these services; and free choice of the insured fund administrator and service provider. It is a variant of managed competition, but it is known in Latin America as structured pluralism^(^ 9 ^)^. This separation is necessary to stimulate market forces and competition, to ideally channel financial resources to the demand, the users, and eliminate the funding of the offer has been suggested, meaning the budget of the public institutional providers of services.

The second innovation is precisely the assurance of 'universal' quality which allows the State to guarantee the public market through insurance, managed by private or public agents, which amounts to a state subsidy to the private sector, as an administrator or service provider^(^ 10 ^)^. The logic of this model is the same as a private insurance, leading to the definition of explicit service packages for each type of insurance. Another way to enforce competition and commodification is with the New Public Management (NPM), with payment to public or private providers on the basis of services actually rendered; which drives outsourcing and job insecurity in the sector^(^ 11 ^)^. 

The best known cases of the UHC via health insurance in Latin America are Chile, Colombia and Mexico. Colombia and Chile implemented them with a comprehensive reform of social security in which health insurance is mandatory, while in Mexico voluntary health insurance with public insurance public social security allowance was added, also reformed to introduce a separation of functions. In all three cases, the existence of fund service managers/buyers and private service providers, as well as freedom of choice of the insured, are promoted and legislated. 

In Chile, the reform led to two parallel systems^(^ 12 ^)^: the private health insurance institutions (ISAPREs) with private providers, and the public National Health Fund (FONASA) with public providers. The health system segmentation was maintained as such and fragmentation of its private component increased. Health packages in the private sector depend on the amount quoted, and high risk people (old or sick people) are excluded; problems that have subsequently limited the establishment of the Explicit Health Guarantees (GES). The FONASA pays, in principle, all of the required services. This led to members of the ISAPREs seeking excluded services from their insurance in the public sector, which resulted in a regressive cross-subsidy; this situation has also been subsequently regulated^(^ 13 ^)^. 

The Colombian reform has another institutional arrangement^(^ 14 ^)^. Simply put, the Solidarity and Guarantee Fund (FOSYGA) receives insurance quotations and allegedly a state subsidy for non-contributors. These financial resources are transferred to managers - the Health Promoting Enterprises (HPE) - which are now mostly private, depending on the number of their policyholders. HPEs administer resources and pay the public and private providers. There are two types of coverage or service packages, one for contributors and one for subsidized policyholders. This arrangement has led to the segmentation and fragmentation of the health system and the public sector has weakened. Public hospitals have even been sold, and the employment situation is increasingly precarious. Implementation of this model has caused many problems^(^ 15 ^)^ in accessing services; with a bureaucracy, sometimes impassable, with differentiated service packages, causing an avalanche of guardianships or injunctions to the judiciary, among others.

Colombia was presented as the success story to follow for nearly two decades. With the declaration of the social health emergency in late 2009,^(^ 16 ^)^ the failure of this reform and economic bankruptcy of the system was revealed. Various corruptions and breaches of payments were also identified, particularly in public hospitals. Its only success had been the generation of high returns for private HPE.

Given the dismal failure of the Colombian model, the Mexican reform^(^ 17 ^)^, particularly the Popular Insurance (SP), is presented today as the success story of the UHCs. There are three forms of insurance in Mexico: the formal social security for workers, the SP for the population without employment insurance, and the private one, without much quantitative importance. Financing is tripartite with an important tax subsidy for the first one, and primarily taxes for the SP, although the payment of a premium by policyholders is suggested. The service packages are different: the SP does not cover more than 20% of social security services and excludes the most costly diseases, treatment is paid by the patient. Both subsystems have their own infrastructure with public providers and paid staff, but may contract with the private sector. Legally, there is no separation of functions between fund management/purchasing services and the rendering of these, but there are no private managers even though there have been attempts to promote them since 1995. 

The reform process has weakened social security, the strongest part of the public health system, but its resistance to private sector attacks is remarkable. It is on the government's agenda to create a "Universal National Health System" (UNHS) through mechanisms of: insurance portability between public and private institutions, with a package of unique services; unique treatment protocols and funded services; and, health market development^(^ 18 ^)^. This approach does not seek to establish a single public health service with universal access, ensuring the right to health. If it materializes, the ones with the biggest loss would be the population with social security, which would have its health benefits significantly reduced; the potential winners would be the private insurers, given the need to purchase insurance that covers illnesses and treatments not included in the basic package. However, the SNSU has not advanced so far because of the lack of fiscal resources and disagreements over financial and institutional design. 

Summarizing the main flaws of the UHC, it is evident that it has not achieved universal insurance coverage; Mexico stands out with 25% of the population having no insurance. Policyholders do not have access to required services because of the restrictions of the service packages covered by their insurance. The confusion in the literature and in the population between insurance coverage and medical coverage or services is apparent; this has led to the belief that having insurance will give one access to all services^1^. The weakening of public services, or their blunt destruction, is another common feature which is explained by the failure to invest in infrastructure and human resources, with the assumption that the private sector and the market will solve the problem. This has occurred despite increases in public health budgets which have been absorbed by private companies or corruption, causing budget crises which suggest further reduction in services.

Although reforms of the UHC type have been successful in their intention to introduce a neoclassical/neoliberal model in the sector, they have caused extensive social rejection. Its promoters have tried to counter this by appropriating the discourse on the right to health and citizenship but, in the social landscape, the barriers outweigh the access to services. Thus, this model tends to delegitimize governments. The tension between the discourse of universality and the reality of the restrictions of services has led to increasing health coverage, but this has not been enough. Additionally, the elements of 'good medicine' are systematically being violated in the interest of profit. Hence the social struggle continues in order to ensure health as a right^(^ 19 ^)^. 

But this model also has built and strengthened private actors in the health sector, and they now have enough economic and political clout to successfully resist the changes they deem contrary to their interests. Thus, the governments are caught between social unrest and resistance to change from their natural allies, the entrepreneurs of national and international health. This classic contradiction of the capitalist State between legitimacy and accumulation has helped lead the government to parties or coalitions that prioritize social welfare and subscribe to the values of the social state, such as in Chile. In other cases, social unrest and general protest against neoliberalism have sparked popular demonstrations which have democratically installed new governments and changed their constitutions, in a process that has led states in transition.

## Health policy of social democratic rule of law or progressive states

The historical trajectory of the progressive governments has been different, but can roughly be grouped into two sets. On one side are the countries that defeated the dictatorships and later chose governments, as a result of popular discontent with the neoliberal policy (Argentina, Brazil, Uruguay, El Salvador, Honduras and Paraguay). On the other hand, we have those where popular mobilizations against the neoliberal model brought the government to its leaders and the passing of new constitutions (Venezuela, Bolivia and Ecuador). These are states in transition, within the meaning of García Linera^(21).^ Progressive governments are characterized by prioritizing social welfare and adopting a comprehensive and redistributive social policy, including the increase of salary or income, job creation and defining the benefits and social services as state responsibilities. This policy involves acting on the social determinants of health-disease. 

In the field of health policy, the Brazilian Constitution of 1988, which states the right to health is an obligation of the state, and it guarantees this through the establishment of one single, free public system (SUS), this has been the paradigm of the new constitutions and the struggles of left-winged parties. Thus, the new constitutions of Venezuela, Bolivia and Ecuador collect this model and also add the concepts of multiculturalism and "Good Living", but have not issued regulatory legislation. The other progressive governments have not made constitutional changes, but have established programs or laws that point in the same direction. For example, the gratuity of services in El Salvador and Paraguay, the Mental Health Act and the REMEDIAR program in Argentina, among others. 

Regardless of their specific legal framework, progressive governments have in common that they have substantially increased access to health services, which is different from simple insurance coverage. For example, coverage increased from 30 to 190 million people with the SUS in Brazil, and 98% of the population could access services when they needed them^(^ 22 ^)^; with Venezuela's Barrio Adentro program, access expanded to 17 million people (57% of the population) who lacked it^(^ 23 ^)^; in Ecuador, access to services and medicines free of charge substantially increased^(^ 24 ^)^; and, Uruguay's new policy has benefited the previously underserved rural population^(^ 25 ^)^.

These achievements are due, first, to a change in the model of care into different forms of Renewed Primary Health Care and Family Medicine, with a special emphasis on education and health promotion without prejudice to preventive, curative and rehabilitative care. On the other hand, they have undertaken a sustained effort to build the infrastructure and train the staff to ensure care, unlike the neoliberal systems which have left this issue to the market. Although it has also been a feature of the health policy based on the UHC, progressive governments have increased the health budget^(^ 26 ^)^. The difference is that progressive governments have strengthened the public sector budget, while in the neoliberal governments the increase has been exploited by the private sector. 

Another important element of health policy is popular and social participation, as it is legally established in Brazil or Venezuela, or as part of the political process such as in Bolivia and Ecuador. Participation has been essential in the design and implementation of the new policy, but like all social and political mobilization, it has had ups and downs. 

## The stresses of progressive health policy

This health policy has produced several effects on society and politics. On the one hand, the health gains of progressive governments have earned them the social recognition of the people. On the other hand, they have aroused higher expectations and demands which stress public institutions and give room for dissent and political struggle. Before analyzing these conflicts, it is necessary to take some considerations into account. 

First, it should be emphasized that a new policy is forged through and with existing institutions, each with its own historical process. This means that one must have an accurate diagnosis of what García Linera^(^ 21 ^)^ calls the institutional materiality, namely, norms, rules and procedures; bureaucracies and hierarchies and habits; and, the budgets. All progressive governments have faced problems which were aroused by this materiality, but have solved them in various ways. Venezuela^(^ 23 ^)^ faced them by creating a parallel health care system, Barrio Adentro (BA), which allowed rapid progress in expanding access and transformation of the care model. However, over time it has become urgent to merge BA and the rest of the public system. Progressive governments, for example Chile and Argentina, with well-established private sector or powerful social work, have proceeded to strengthen state regulation and public institutions^(^ 27 ^)^. In Brazil, the near absence of a public system facilitated the task initially but left legal loopholes which were exploited by the private sector to expand and strengthen itself ^(^ 28 ^)^. It therefore seems crucial to have a strategic plan to make decisions to solve problems without violating the ultimate goal of building a unique and supportive public health system.

Another crucial issue is that, in countries with progressive governments, the conception of health as a right that is an obligation of the State has become a widely appreciated social value, thanks to the action of the governments themselves. However, this has not necessarily led to a comprehensive and social understanding of health. This means that the medicalized idea of 'good medicine' often prevails. This is crucial for the possible actions that governments can propose. If health authorities have failed to position the integral and social understanding of health before the public and the rest of the government, there is a risk of treating the problem as a technical issue, and allocating financial resources without analyzing the best way to address the health needs of the population. The problem is not trivial because it is technical-financial and conceptual at the same time. 

Latin American health systems, including in the progressive countries, often lack the necessary expertise competence and it needs urgent development. They also all tend to be sub-funded and require more budgetary resources. However, the conception of health-disease and its social determination is crucial when making decisions on priorities to guide technical-scientific development, and to calculate the required financial resources. No one denies the need to provide quality and technically satisfactory services. It is in this context that we should settle the case for a single public health system, which is the most suitable and inexpensive institutional arrangement to respond to health needs, but also to combat the commoditization and dehumanization of health^(^ 29 ^)^. 

In the budgetary process that includes other social areas, it seems insufficient to consider health as just one more social right. It may be helpful to take up the idea of positive freedoms, which are those that allow full participation in a democratic society. One is health as a basic human need,^(^ 30 ^)^ the satisfaction of which is essential for such participation. Also, it reinforces the idea that health is not an object of consumption^(^ 1 ^)^ but a means to develop skills and potentials as well as individual and collective autonomy. 

The misunderstanding of these tensions or shortsighted wrong decisions can lead to health actions becoming an ingredient in a process of delegitimization or questioning of the government, even though they initially helped to legitimize it. The fact that health is also an important area of capital accumulation in post-neoliberal societies plays an important role. Thus, members of the medical-industrial-insurer complex exercise all their influence to assert their interests as sellers of medications, supplies, medical equipment and insurance. They are the first to say that public institutions offer "poor medicine for poor people". The best strategy to counter this argument is to strengthen and enhance institutional capacity by providing technically competent and humanly satisfactory public services that displace private services. 

This is not enough if the ideological content of 'good medicine' and its articulation with the capital accumulation is not revealed. There are many elements needed to do this, because there is extensive literature on the abuse and damage caused by the desire for gaining from the medical-industrial complex. In this context, when strengthening state regulation, technology assessment, production of medicines and other supplies is also crucial. 

Another key issue is facing the insurance or private health plans that persist and even grow in the conceptually unique and public health systems^(24, 31-32).^ This is necessary because they channel significant amounts of public resources into private ones in various ways that weaken the public system^(^ 26 ^)^. A paradoxical obstacle is that employment benefits are usually negotiated by the large unions, meaning the natural class basis of the public, solidarity and egalitarian systems^(^ 32 ^)^. Thus there is the governmental temptation to encourage private insurance, arguing that decompressed demand in the public system is equivalent to naturalizing inequality in access to required services, especially when the door opens to large corporations of international health. The most effective antidote is informed popular participation, which promotes political-ideological and cultural change. Another temptation is to adhere to the model of 'universal' assurance that, as discussed above, means that the private use of public resources for the sake of unproven ideological premise.

## Conclusion

Health reforms in Latin America are taking place in two opposing ways: the UHC and the SUS. They are inserted into two different developmental models which are in the composition and role of the state in economic and social policy. Neoliberal governments have adopted the neoclassical economic thought, and consider health a field of free market economy. The UHC, through health insurance, is the health policy that has strengthened the medical-industrial-insurer complex and increased profits, but at the expense of universal and equal access to health services and governmental legitimacy.

Progressive governments have increased access and guaranteed the right to health through their unique, public and supportive health systems, but they face several challenges related to growing demands of the population and the persistence of an aggressive private sector.

## Footnotes

^1^The most famous is the disputed WHO 2000 Report which launched a method to measure performance, in which the percentage of policyholders was given a very important role^(20)^
